# Cost-effectiveness analysis of azacitidine in the treatment of high-risk myelodysplastic syndromes in Spain

**DOI:** 10.1186/2191-1991-3-28

**Published:** 2013-12-05

**Authors:** Carlos Crespo, Estela Moreno, Jordi Sierra, Suzan Serip, Marta Rubio

**Affiliations:** 1 Statistic Department, University of Barcelona, C/Diagonal 643, Barcelona 08028, Spain; 2 Health Economics & Outcome Strategies Department, Oblikue Consulting, C/Josep Irla i Bosch 5-7, Barcelona 08034, Spain; 3 Hospital de Santa Creu i Sant Pau, c/Sant Antoni Maria Claret 167, Barcelona 08025, Spain; 4 Market Access, Celgene, Paseo de Recoletos 37-39 4a, Madrid 28004, Spain

**Keywords:** Cost-effectiveness, Myelodysplastic syndrome, Azacitidine, Chemotherapy, Best supportive care

## Abstract

**Background:**

The objective of the study was to analyse whether azacitidine is a cost-effective option for the treatment of myelodysplastic syndrome in the Spanish setting compared with conventional care regimens, including best supportive care, low dose chemotherapy and standard dose chemotherapy.

**Methods:**

A life-time Markov model was constructed to evaluate the cost-effectiveness of azacitidine compared with conventional care regimens. The health states modelled were: myelodysplastic syndrome, acute myeloid leukemia and death. Variables measured included survival rates, progression probabilities and quality of life indicators. Resource use and cost data reflect the Spanish context. The analysis was performed from the Spanish National Health System perspective, discounting both costs (in 2012 euros) and future effects at 3%. The time horizon considered was end-of-life. Results were expressed in cost per quality-adjusted life-year gained and cost per life-year gained and compared with cost-effectiveness thresholds.

**Results:**

According to the current use of each conventional care regimens options in Spain, azacitidine resulted in €34,673 per quality-adjusted life-year gained (€28,891 per life-year gained) with an increase of 1.89 in quality-adjusted life-years (2.26 in life-years). Azacitidine was superior to best supportive care and low dose chemotherapy in terms of quality-adjusted life-years gained, 1.82 and 2.03, respectively (life-years 2.16 *vs.* best supportive care, 2.39 *vs.* low dose chemotherapy). Treatment with azacitidine resulted in longer survival time and thus longer treatment time and lifetime costs. The incremental cost-effectiveness ratio was €39,610 per quality-adjusted life-year gained *vs.* best supportive care and €30,531 per quality-adjusted life-year gained *vs.* low dose chemotherapy (€33,111 per life-year gained *vs.* best supportive care and €25,953 per life-year gained *vs.* low dose chemotherapy).

**Conclusions:**

The analysis showed that the use of azacitidine in the treatment of high-risk myelodysplastic syndrome is a cost-effective option compared with conventional care regimen options used in the Spanish setting and had an incremental cost-effectiveness ratio within the range of the thresholds accepted by health authorities.

## Background

Myelodysplastic syndrome (MDS) is a group of medical conditions derived from progressive bone marrow failure that result in ineffective production of blood cells. Depending on the severity, MDS reduces the quality of life to the point of being life-threatening. There is a probability of death at all stages of the disease, due to complications and co-morbidities, with progression to acute myeloid leukaemia (AML) being the worst evolution [1]. Together with advanced age, exposure to tobacco and some chemical agents and previous chemotherapy as treatment for non-related diseases are potential risk factors [1]. The International Prognostic Scoring System (IPSS) identified three critical factors that influence survival and AML evolution: risk-based cytogenetic subgroups (good, intermediate and poor karyotypes), bone marrow blast percentage and the number of cytopenias. According to these factors, IPSS groups include patients in four risk categories. The low and intermediate-1 groups are described as lower-risk patients associated with longer median survival and time to progression to AML; the intermediate-2 and high groups, in contrast, are associated with poor median survival and shorter median time to progression to AML [2].

MDS patients have a 20-30% probability of progression to AML and a 40-65% probability of death due to complications and co-morbidities, with the frequency depending on age and comorbidities [1]. Therefore, a careful differential diagnosis is required for rapid identification and treatment of the disease.

The conventional care regimen (CCR) for high-risk MDS is best supportive care (BSC), low-dose chemotherapy (LDC) or standard dose chemotherapy (SDC) [3]. Treatments are associated with a survival rate of 1.2 years for intermediate-2 stage MDS and 0.4 years for high [2]. BSC is a common choice of treatment for high-risk MDS patients but is only palliative and no improvement in overall survival (OS) or progression to AML has been shown compared with LDC which, despite its clinical benefits is associated with potentially high infection rates [4]. SDC is associated with high mortality (<35%), short duration of remission (usually less than 12 months), prolonged hospitalisation and a significant reduction in quality of life [5-7]. Allogeneic stem cell transplantation is the only potentially curative treatment available but is only feasible in 5% of patients depending on the availability of a suitable donor, age and comorbidities [8]. Several studies have compared azacitidine to current treatment options and have shown large improvements in survival and quality of life. AZA-001, an international, multicentre, controlled, open label, randomised, parallel-group, comparative phase III study showed a significant median gain in OS of 9.4 months (12.9 *vs.* BSC, 9.1 *vs.* LDC and 8.7 *vs.* SDC) [9]. The CALGB 9221, a prospective, open label, multicentre, randomised, controlled phase III study conducted by the Cancer and Leukemia Group B (CALGB) confirmed a median OS increase in survival of 8.5 months and a statistically significant improvement in physical functioning, fatigue and dyspnoea [10].

Spanish guidelines recommend azacitidine in the treatment of patients who are not eligible for haematopoietic stem cell transplantation with IPSS intermediate-2 and high-risk MDS and patients with low-risk MDS after failure of erythropoiesis-stimulating agents and patients with chromosome 5q deletion MDS after lenalidomide failure [11].

Positive clinical results require economic evaluation in order to make appropriate healthcare decisions on cost and resource use. The objective of this study was to assess whether azacitidine is a cost-effective treatment from the Spanish health perspective compared with CCR options.

## Methods

Azacitidine was compared with CCR treatment options using a cost-effectiveness economic analysis based on a life-time Markov model.

**Figure 1:**
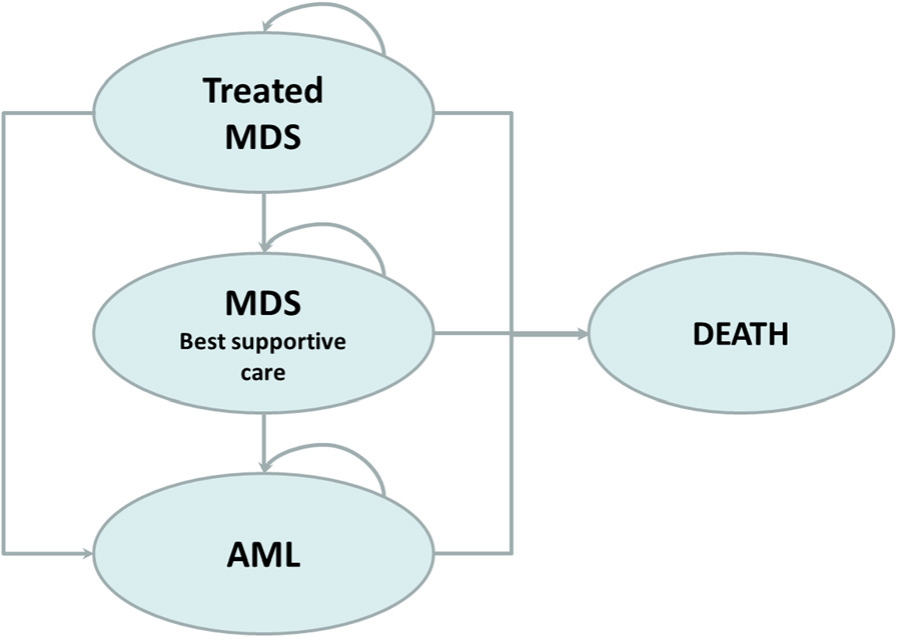
**Markov model structure.** MDS = myelodysplastic syndrome; AML = acute myeloid leukaemia.

The model simulated MDS management by assigning cost and health values to the transition probabilities of three mutually exclusive health states arising from the evolution of MDS over a life-time period. Patients were assumed to start in the MDS state and receive first-line treatment (azacitidine, BSC, LDC or SDC) and then either die or progress to AML with consequent progression to death. Once they progressed to AML they only received BSC. The health states modelled were MDS with/without treatment, AML and death. Survival rates, progression probabilities and quality of life indicators were measured (Figure 1).

A MEDLINE literature search was carried out to obtain data up to June 2012 on the efficacy of azacitidine and comparators using the keywords: azacitidine, high-risk myelodysplastic syndrome and phase III clinical trial. Articles referring to comparators not indicated for the treatment of high-risk MDS or which are not licensed in Spain, were excluded. The efficacy data used in the model was taken from the AZA-001 randomized clinical trial which included 358 high-risk MDS patients who received azacitidine, BSC, LDC or SDC [9]. Median OS and the median time to progress to AML were the main efficacy results assessed in the study, while safety results referred mainly to adverse events (AE).

To estimate survival beyond that observed in the AZA-001 trial, the adjustment of the survival curves to different probability distributions (Weibull, exponential, log-normal and logistic) was analysed using statistical techniques.

The distribution selected was that which best fit the observed data. Finally, the 2-year survival curves for each treatment arm were extrapolated using the log-normal distribution. The model also considered treatment cessation for each treatment arm, which was extrapolated in the same manner as survival, as well as the probability of progression to AML. The mortality rate from AML was assumed to be the same for all treatment arms: 0.135 per 5-week cycle [9].

Utility scores were introduced into the model to assess patients’ preferences for the health outcomes and build the result variable, quality-adjusted life year (QALY). Utility scores are measured on an interval scale with zero representing health states equivalent to death and one representing perfect health. When generic utility scores (EQ-5D) were not available, a mapping procedure was used. MDS and BSC utility scores were mapped to translate the European Organization for Research and Treatment of Cancer (EORTC QLQ-C30) scores from the CALGB study to EQ-5D scores using regression analysis [12,13]. SF-12 utility scores for LDC and SDC [7] were mapped to EQ-5D values using regression analysis and Monte Carlo simulation [14].

Quality of life was similar for all treatment arms: the baseline utility score in the MDS state was 0.67 for azacitidine, BSC and LDC and 0.66 for SDC [13]. Patients with AML had a worse quality of life than those with MDS, which had a utility score of 0.52 [15]. As the CALGB study only considered the quality of life of patients with MDS up to 182 days and in the absence of long term evidence of quality of life with MDS, it was assumed that this would remain constant during the follow-up period.

All available health state management costs per unit were adapted from the Spanish Cost Database [16] (Table 1) and pharmaceutical costs were taken from a specific local database [17] (Table 2). The model assumed wastage for all pharmacological options. Costs were expressed in 2012 euros and costs and effects were discounted by 3% over a life-time horizon.

**Table 1:** Unit costs of resources

**Resources**	**Cost per unit**	**Description**
Inpatient hospital stay (€ per days)	€742.91	standard length of stay 28 days
Haematologist	€62.22	Standard MDS visit
Nurse	€33.20	Average cost
**Test**		
Biochemical Profile	€44.03	Average cost
Bone Marrow (Aspirate)	€133.80	Average cost
Full Blood Count	€6.08	Average cost
**Transfusions**		
Platelet transfusion	€352.81	
Blood transfusion	€353.23	
**Adverse Events**		
Neutropenia	€68	Medical visit and analytics
Leucopenia	€68	Medical visit and analytics
Febrile neutropenia	€3,735	RDG 722. Simple pneumonia and pleurisy
Pyrexia	€3,735	RDG 722. Simple pneumonia and pleurisy
Pneumonia	€3,735	RDG 722. Simple pneumonia and pleurisy
Sepsis	€3.728	ICD 205.00

Abbreviations: MDS = myelodysplastic syndrome; DRG = Diagnosis-Related Group; ICD = International Classification of Diseases.

**Table 2:** Pharmacological pattern and cost per cycle

**Treatment**	Cost/mg^**^	**Dosage/(mg/m**^**2**^**)†**	**Days of treatment/cycle**	**Cost per cycle**
**AZA REGIMEN**				
**Azacitidine**	€3.40/mg	75	7	**€3,028.14**
**LDC REGIMEN**				
**Cytarabine**	€0.0271/mg	150	7	**€48.38**
SDC REGIMEN*				
**Cytarabine/Idarubicin**				**€965.16**
Cytarabine	€0.0271/mg	1000	7	€322.56
Idarubicin	€10.52/mg	12	3	€642.60
**Cytarabine/Mitoxantrone**				**€543.55**
Cytarabine	€0.0271/mg	1000	7	€322.56
Mitoxantrone	€3.62/mg	12	3	€220.99
**Cytarabine/Daunorubicin**				**€377.12**
Cytarabine	€0.0271/mg	1000	7	€322.56
Daunorubicin	€0.179/mg	60	3	€54.56
**Cytarabine/Idarubicin/Etoposide**				**€848.68**
Cytarabine	€0.0271/mg	1000	4	€184.32
Idarubicin	€10.52/mg	12	3	€642.60
Etoposide	€0.043/mg	100	3	€21.76

Abbreviations: AZA = azacitidine; LDC = low dose chemotherapy; SDC = standard dose chemotherapy.

* Data were pooled corresponding to Spanish usual care weight for each treatment (pooled cost: €790. 41). ** All costs are expressed in ex-factory price and are discounted according to RD 15/2010 † Mean body surface was assumed to be 1.7 m^2^.

The burden per cycle included both MDS on and off treatment costs and AML-related expenses. Untreated MDS and AML reflected the cost of BSC with a different resource use pattern, while treated MDS adds pharmacological treatment and its administration cost (Table 3). Follow-up appointments represent routine haematologist and nurse visits and were the same for all MDS patients regardless of the type of treatment (2 haematologist and 2 nurse visits). However, patients in AML state only attended 3 haematologist visits. The typical routine tests applied for assessing MDS disease evolution are: biochemistry profile, full blood count and bone marrow aspiration. There are only slight differences between the cost of routine tests in AML and MDS off treatment state due to fewer full blood count tests (2 *vs.* 1) but there are large differences in medication options, mainly due to the bone marrow aspiration test needed for pharmacological therapy. Concurrent medication also varied depending on whether the patients were on/off pharmacological treatment or had AML, which resulted in greater costs for AML than for the other states. A large part of the total cost was due to transfusions, which included blood and platelet transfusions, and varied according to the treatment arm depending on the number of units administrated in the AZA-001 study (the cost for AML was assumed to be equal to the cost for BSC) (Table 3).

**Table 3:** Treatment costs per cycle

	**Azacitidine**	**BSC**	**LDC**	**SDC**
**MDS treatment on treatment**	**€4,911.24**	**€1,426.21**	**€2,671.20**	**€20,853.08**
Pre-medication	€0.70	€0	€2.11	€0
Treatment administration	€442.40	€0	€380.20	16,344.02
Pharmacology	€3,028.14	€0	€48.38	€790.41
Follow-up appointments	€238.55	€238.55	€238.55	€0^*^
Blood/Platelet transfusion	€926.07	€1,070.31	€1,754.35	€2,557.71
Concurrent Medication on treatment	€37.90	€54.72	€65.86	€87.21
Routine tests on treatment	€237.49	€62.63	€181.74	€1.073.74
**MDS treatment off treatment**	**€1,627.78**	**€1,772.02**	**€2,456.06**	**€3,259.42**
Follow-up appointments	€238.55	€238.55	€238.55	€238.55
Blood/Platelet transfusion	€926.07	€1,070.31	€1,754.35	€2,557.71
Concurrent Medication off treatment	€54.72	€54.72	€54.72	€54.72
Routine tests off treatment	€62.63	€62.63	€62.63	€62.63
Annualized Adverse Events BSC	€345.81	€345.81	€345.81	€345.81
Treatment administration BSC	€0	€0	€0	€0
**AML treatment**	**€1,851.86**	**€1,851.86**	**€1,851.86**	**€1,851.86**
Follow-up appointments	€233.33	€233.33	€233.33	€233.33
Adverse events	€345.81	€345.81	€345.81	€345.81
Concurrent Medication	€132.18	€132.18	€132.18	€132.18
Blood/Platelet transfusion	€1,070.31	€1,070.31	€1,070.31	€1,070.31
Routine tests	€70.24	€70.24	€70.24	€70.24

Abbreviations: MDS = myelodysplastic syndrome; AML = acute myeloid leukaemia; BSC = best supportive care; LDC = low dose chemotherapy; SDC = standard dose chemotherapy.

* Include only 1 chemotherapy session.

In patients on treatment, AE were modelled using AZA-001 annualized clinical trial data and the AE rate per five-week cycle was calculated. In patients off treatment, the annualized AE rate for BSC was used. AE costs for each treatment arm were calculated by multiplying local AE resource use cost data [16] by the AE rates obtained.

To obtain the necessary inputs and arrive at a consensus on resource use, two medical specialists, one hospital pharmacist and one haematologist participated in two rounds of independently-answered clinical surveys. All unit costs and results were validated by this expert group.

In terms of clinical benefits, results were expressed as life-years (LYs) gained and QALYs gained. From the cost perspective, the total cost of each alternative and the cost per cycle were compared. In terms of cost-effectiveness, the incremental cost per LY gained and QALY gained was compared with cost-effectiveness thresholds.

$$\frac{{\text{Cost}}_{\text{azacitidine}}\;-\;{\text{Cost}}_{\text{CCR}\;}}{{\text{Effectiveness}}_{\text{azacitidine}}\;-\;{\text{Effectiveness}}_{\text{CCR}\;}}$$

A cost-effectiveness threshold is the amount of money the decision maker is willing to pay for each LY or QALY gained. Due to the fact that there is no fixed value and to the lack of consensus in Spain, a threshold of €50,000 per QALY gained, for end-of-life drugs, was used according to the UK National Institute for Health and Clinical Excellence [18,19].

The analysis was conducted taking into account the typology of the patients and therefore treatment assignation to homogenous groups of patients was made according this typology. In the AZA-001 trial, azacitidine was administered to 110 patients, BSC to 79, LDC to 38 and SDC to 20 [9]. Our study replicated this treatment pattern: BSC with blood product transfusions and antibiotics with granulocyte colony-stimulating factor for neutropenic infection; LDC with cytarabine, 150 mg/m^2^ per day subcutaneously for 7 days, every 28 days for at least 4 cycles; and SDC with cytarabine 1000 mg/m^2^ per day for 7 days, plus 3 days of either intravenous daunorubicin [60 mg/m^2^ per day], idarubicin [12 mg/m^2^ per day] or mitoxantrone [12 mg/m^2^ per day]) or 3 days of idarubicin [12 mg/m^2^ per day] and etoposide [100 mg/m^2^ per day]. For a more exact approximation to the Spanish context, the expert group suggested only including one session of SDC treatment in the analysis, despite the fact that in the AZA-001 trial patients received a median of one session.

A global cost-effectiveness analysis of azacitidine *vs.* BSC, LDC and SDC and a sub-analysis of azacitidine compared with BSC and azacitidine compared with LDC were made. The results of an analysis for azacitidine compared only with SDC were considered not applicable due to the low number of patients enrolled (low power for small size samples).

Probabilistic sensitivity analysis was performed to examine the combined effect of the uncertainty in all the variable parameters (survival, treatment cessation, unit cost, use of resources, etc.). Values were sampled from the uncertainty distributions associated with each parameter. Where there were no estimates of parameter uncertainty, ±30% intervals were assumed. To achieve this, results were generated for a hypothetical sample of 50,000 individuals using a parametric Monte-Carlo simulation based on the variability in the curve fit and extrapolation in the efficacy and on the range of costs (maximum and minimum) of the resources used. The log-normal distribution was used for survival data, a Weibull distribution for treatment cessation, a beta distribution for utilities and AE and a normal distribution for dosing and resource use [20,21]. Uncertainty in the survival and treatment cessation variables was analyzed by incorporating the covariance generated in the survival models [20,21].

## Results

**Figure 2:**
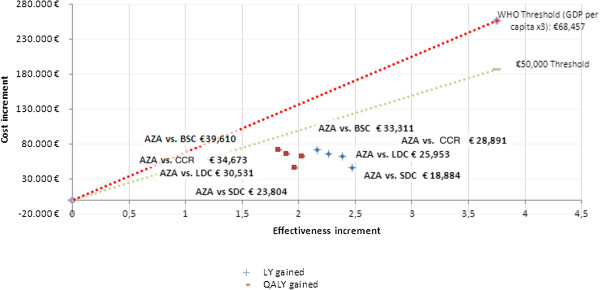
**Cost-effectiveness plane AZA vs. BSC and AZA vs. LDC and AZA vs. CCR.** AZA = azacitidine; BSC = best supportive care; LDC = low dose chemotherapy; SDC = standard dose chemotherapy; CCR = conventional care regimen; QALY = quality-adjusted life year; LY = life year; GDP = Gross domestic product.

A cost-effectiveness analysis was performed to analyse azacitidine *vs.* the three CCR options assessed in the AZA-001 clinical trial (BSC, LDC and SDC). The analysis for the lifetime perspective of azacitidine *vs.* the weighted mean survival of the CCR options showed 2.26 LY gained and 1.89 QALY gained. The survival gained with azacitidine resulted in longer treatment time and thus greater accumulated costs over a life-time horizon, resulting in higher costs *vs.* the CCR options (€65,436) (Table 4). Furthermore, the ICER value was €28,891/LY gained and €34,673/QALY gained and was located under the €50,000/QALY cost-effectiveness threshold (Figure 2).

**Table 4:** Cost-effectiveness analysis results over a life-time horizon

**Indicator**	**Costs**		**LYs**		**QALYs**	
Treatment	AZA	comparator	AZA	comparator	AZA	comparator
**AZA** ***vs.*** **BSC**	**€**107,168	**€**35,090	4.05	1.88	3.06	1.24
ICER			**€33,111/LY gained**		**€ 39,610/QALY gained**	
**AZA** ***vs.*** **LDC**	**€**115,537	**€**53,184	4.45	2.06	3.39	1.36
ICER			**€25,953/LY gained**		**€30,531/QALY gained**	
**AZA** ***vs.*** **SDC**	**€**106,422	**€**59,725	3.96	1.49	2.94	0.98
ICER			**€18,884/LY gained**		**€23,804/QALY gained**	
**AZA** ***vs.*** CCR*	**€**108,605	**€**43,170	4.11	1.85	3.11	1.22
ICER			**€28,891/LY gained**		**€34,673/QALY gained**	

Abbreviations: AZA = Azacitidine; BSC = best supportive care; LDC = low dose chemotherapy; SDC = standard dose chemotherapy; CCR = conventional care regimen; QALY = Quality adjusted life year; LY = Life year.

* Data were pooled corresponding to the number of patients in the AZA-001 study for each treatment.

Moreover, a sub-analysis of azacitidine compared with BSC and LDC was also performed in order to show the differences between the subgroups included in the analysis.

### Azacitidine vs. BSC

Azacitidine showed greater clinical benefit over a life-time horizon compared with BSC. While azacitidine added 4.05 years, BSC added only 1.88 years. Moreover, azacitidine improved the quality of life, with 3.06 QALY gained compared with 1.24 QALY gained using BSC.

**Figure 3:**
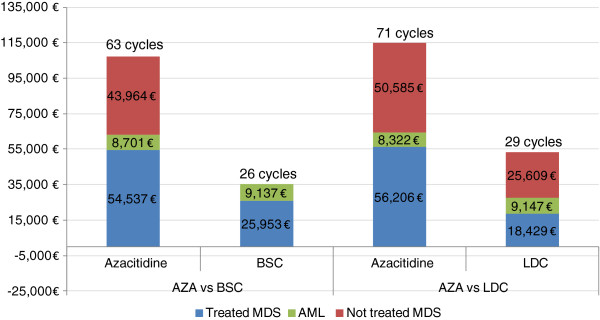
**Cost of treatments and overall survival (treatment cycles).** MDS = myelodysplastic syndrome; AML = acute myeloid leukaemia; AZA = Azacitidine; BSC = best supportive care; LDC = low dose chemotherapy.

Better overall survival with azacitidine resulted in longer treatment time and partially explained the greater accumulated costs over a life-time horizon (Figure 3). The contribution of the MDS off-treatment cost in lifetime treatment burden was noteworthy.

**Figure 4:**
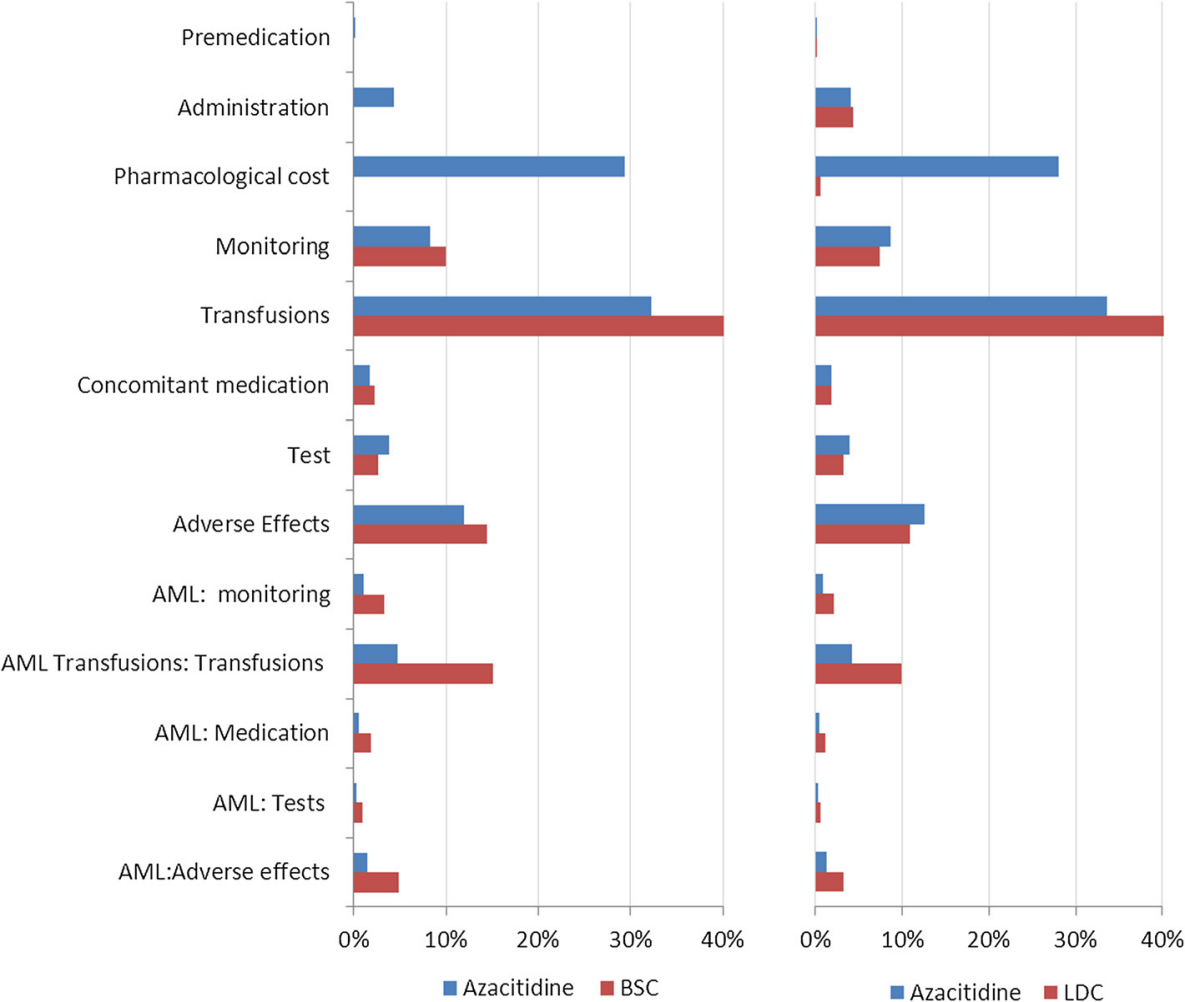
**Cost structure (%) of each treatment arm.** AML = acute myeloid leukaemia; BSC = best supportive care; LDC = low dose chemotherapy.

One cycle of azacitidine cost €4,911 compared with €1,772 for BSC, although the difference was partially compensated for by lower AE costs (€330 *vs.* €345). From the life-time perspective, the total cost of azacitidine was €72,112 higher than the cost of BSC, mainly due to the higher pharmacological cost of azacitidine. However, the reduction in the number of transfusions, representing 32% and 45% of the entire treatment cost of azacitidine and BSC, respectively, partly compensated for the acquisition cost of the drug (Figure 4).

**Figure 5:**
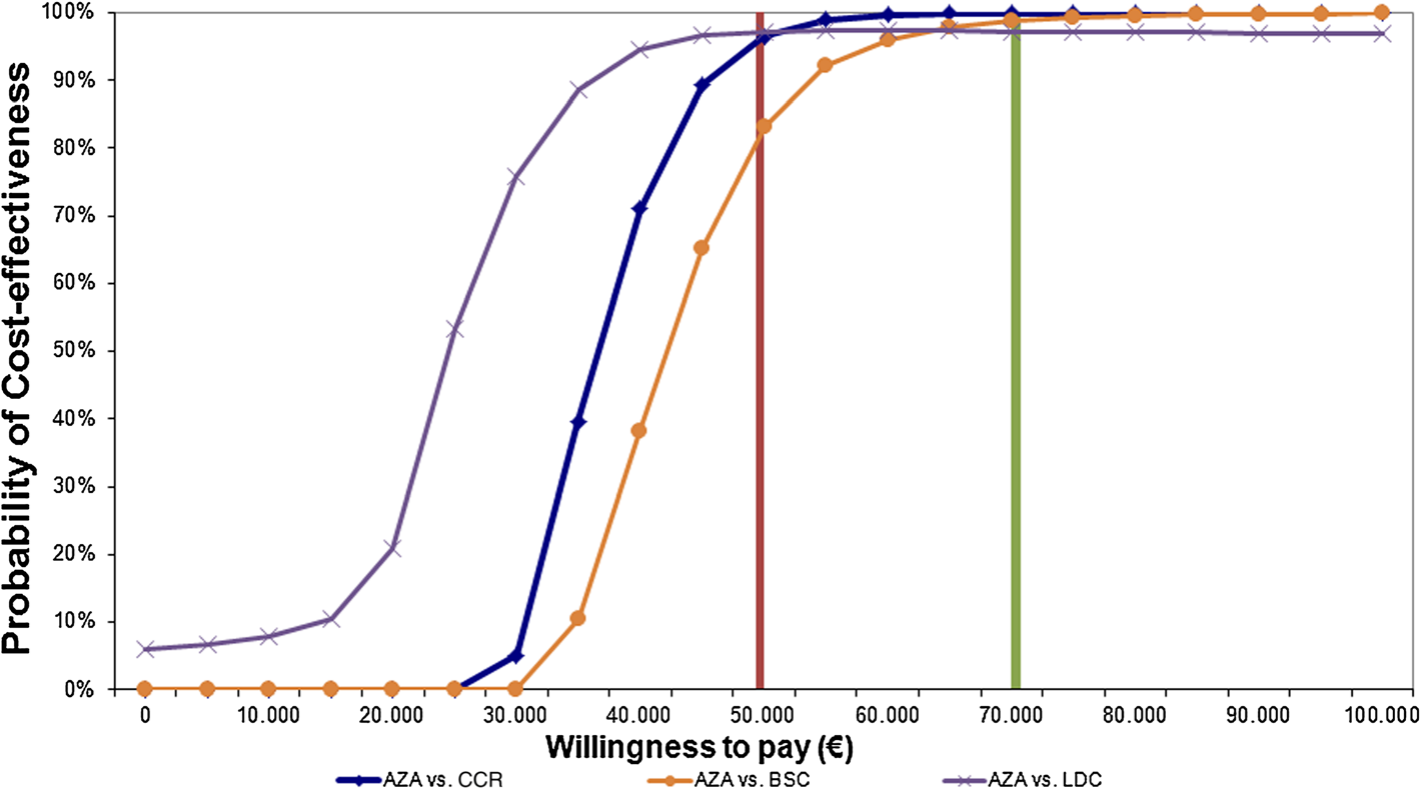
**Cost-effectiveness acceptability curves: Azacitidine vs. BSC and Azacitidine vs. LDC.** AZA = azacitidine; BSC = best supportive care; LDC = low dose chemotherapy; CCR = conventional care regimen.

In terms of cost-effectiveness over a life-time horizon, the incremental cost of azacitidine treatment was €33,111/LY gained. When quality of life was taken into account, the ICER was €39,610/QALY gained. Therefore, azacitidine was considered a cost-effective option in the Spanish setting due to the fact that the ICER value was situated under the €50,000/QALY cost-effectiveness threshold (Figure 5).

### Azacitidine vs. LDC

The same trend was observed in the comparison with LDC. Azacitidine resulted in 2.39 more LY gained and 2.02 more QALY gained compared with LDC. A cycle of treatment with azacitidine cost €4,911 compared with €2,671 for LDC but was partially compensated for by lower AE costs (€330 *vs.* €627). From the life-time perspective, the total cost of azacitidine was €61,929 higher than LDC. As with BSC, the main cost driver of the LDC arm was transfusions (Figure 4).

In terms of cost-effectiveness over a life-time horizon, the incremental cost of azacitidine treatment was €25,953/LY gained and €30,531/QALY gained, values located below €50,000/QALY cost-effectiveness threshold (Figure 2).

### Azacitidine vs. SDC

Due to the low number of patients included in the SDC treatment arm in the AZA-001 trial, this data was used only in the overall analysis of the CCR options. The SDC treatment cost/cycle was almost four times more expensive than azacitidine. The main cost driver of SDC treatment was the administration cost, due to hospitalization in patients receiving this treatment (28 days). There were also significantly greater AE costs compared with azacitidine.

### Sensitivity Analysis

The sensitivity analysis showed that azacitidine was a cost-effective option in 96.49% of the simulated cases €50,000/QALY willingness-to-pay. In the subgroup analysis, the comparison shows that the probability of azacitidine being cost-effective below the €50,000/QALY threshold was 83.21% *vs.* BSC and 91.21% *vs.* LDC (Figure 5). The probabilistic sensitivity analysis confirmed the robustness of the results of the model.

## Discussion

The crude incidence rate of MDS in Spain is 8.1/100,000 [22]. According to the European Medicines Agency (EMA) MDS is considered an orphan disease [23]. Life-threatening diseases with a prevalence rate lower than 5/10,000 are considered rare diseases [24]. Azacitidine was granted orphan drug status in the EU for the treatment of MDS in February 2002 and for the treatment of AML with 20-30% blasts in the marrow in November 2007. Orphan drugs frequently present ICER values far above established cost-effectiveness thresholds, but this is not the case with azacitidine, which has a low ICER value compared with other orphan drugs [23-28]. At present, there is no accepted cost-effectiveness threshold for this type of extreme clinical situation. Nevertheless, even if the choice of the €50,000/QALY threshold might be questioned, it is within the range used in similar studies [19,25-30]. Furthermore, according to the recommendations of the Commission on Macroeconomics and Health Gross Domestic Product (GDP)-based threshold of the World Health Organisation, the maximum threshold would be €68,457/QALY (3xGDP/per capita) [31].

European and Spanish health authorities promote the investigation and development of this type of drug. According to the Spanish Ministry of Health, Social Services and Equality, orphan drugs are “medicines that for economic reasons are unlikely to be investigated and supported by pharmaceutical companies”. Furthermore, in recent years, 87% of orphan drugs positively evaluated by the EMA were licensed in Spain. Most were for use in oncology or endocrinology and metabolism-related diseases [32].

Several studies have assessed the clinical benefits of azacitidine compared with CCR and have shown clinical superiority, but economic evaluations are lacking. A Canadian study comparing azacitidine with CCR options (BSC, LDC, SDC) confirmed the superiority of azacitidine in terms of cost-effectiveness, with a global ICER of CAD 84,395/QALY gained, which was below the 3xGDP WHO threshold (38,710 × 3) [19] for Canada. The study also showed the superiority of azacitidine compared with each of the other treatment options, with the following ICER values: CAD 84,395/QALY gained for BSC, CAD 88,786/QALY gained for LDC and CAD 28,501/QALY gained for SDC [33]. Though not included in our analysis due to the small number of patients participating in the AZA-001 study, the results of SDC treatment in Spain confirmed the trend shown in the Canadian study of an ICER ratio lower than those of the other CCR options.

A recent study compared azacitidine with decitabine and found that azacitidine was a cost-effective treatment for MDS according to U.S. National Healthcare Input data [34], with a comparative gain of 0.171 more QALYs and savings of €15,890 over a 2-year period. However, the relevance of the study is limited, mainly because survival data was retrieved from two different phase III trials and no direct comparison was made.

This study nevertheless has some limitations. It is recommended to consider the fact that this approach is based on a mathematical model which depends mainly on the accuracy of available data in the moment of the analysis and should be treated as it.

To obtain a global cost-effectiveness analysis, a weighted average of individual cost-effectiveness ratios was used. In the absence of local patient treatment distribution data, the ICERs were weighted according to the distribution of patients for each treatment arm in the AZA-001 trial.

In the AZA-001 study, no significant difference between azacitidine and SDC was found [9], and thus the cost-effectiveness analysis for this group might also not be significant.

The cost and resource data used in the model were the best available and came from published data in Spain, obtained from clinical trials, local databases and relevant literature. However, the data sources for efficiency had limitations derived from the limitations, structure and temporality of the trials. In the absence of end-of-life survival data from the AZA-001 study, survival curves were extrapolated using the log-normal distribution. Considering that major clinical benefits are visible in the long term and that our results are difficult to collate due to the fact that MDS is an orphan disease, the clinical experts agreed on the reasonableness of our findings.

However, there is uncertainty about the information on the quality of life as mapping using regression was used to obtain EQ-5D utility scores from the QLQ-C30 and SF-12 scores. Another limitation of utility scores is that QLQ-C30 scores do not include information on fatigue, although this may be implicit in the ability to carry out the activities of daily living. Statistical analysis was used to reduce limitations due to data variability by means of sensitivity analysis, which is used to assess uncertainty by assigning specific distribution probabilities to all data included in the model in order to simulate alternative values. For further analysis, improvements in the quality of life due to the independence of blood transfusion may be included. Likewise, azacitidine may be indicated in cases where blood transfusion is rejected for religious reasons.

Though economic analysis is an important factor in decision making, the clinical perspective remains crucial, and from this perspective, although it is a serious, life-threatening disease, MDS has a high unmet diagnosis and treatment need.

First, due to differences in diagnostic methods and classification, current prevalence rates around Europe are lacking [35]. Secondly, at present, the lack of standardization of MDS in Europe leads to difficulties in determining the appropriate treatment. Thirdly, CCR options are often associated with significant toxicity and morbidity. Azacitidine, on the other hand, showed clinical superiority in terms of outcomes and improvements in safety compared with conventional treatment alternatives [9].

## Conclusions

There is an urgent need for better and safer treatment options for MDS. Azacitidine showed clinical superiority to all of the treatment alternatives considered and the cost-effectiveness analysis showed that azacitidine is a cost-effective treatment option in the Spanish context. End-of-life economic analysis assigned a higher cost to azacitidine treatment partly due to the greater best supportive care costs associated with longer survival.

## Abbreviations

MDS: Myelodysplastic syndrome; AML: Acute myeloid leukaemia; IPSS: International Prognostic Scoring System; CCR: Conventional care regimen; BSC: Best supportive care; LDC: Low-dose chemotherapy; SDC: Standard dose chemotherapy; OS: Overall survival; CALGB: Cancer and leukemia group B; QALY: Quality adjusted life year; EMA: European medicines agency; EORTC QLQ-C30: European organization for research and treatment of cancer; AE: Adverse events; LY: Life year; GDP: Gross domestic product; DRG: Diagnosis-Related Group; ICD: International Classification of Diseases.

## Competing interests

Dr. EM and Dr. JS received consulting fees from Celgene. CC and SS worked in an independent consultant company and received funds from Celgene. MR is a former Celgene employee.

## Author contributions

MR developed the idea for the study, supervised the whole study, and was involved in its design. Dr. EM and Dr. JS provided background information based on their experience as experts and investigators on this field. CC was involved in the study design, carried out the research, the data analysis and drafted the report. All the investigators contributed to the final version of the report. All authors read and approved the final manuscript.
